# 

**Published:** 1915-11-20

**Authors:** 


### CONTENTS.

PAGE

**Instruction in Medical Ethics** ... ... ... 153

**The Right to Change Your Doctor** ... ... 154

**Hospital and Institutional News ...** ... ... 155

St. Bartholomew's Hospital—The Enlargement of the Royal Red Cross—The Planning of Base Hospitals in France—A Three Years' Dispute for Nothing—The British Red Cross Society in Spain—Reduction of Sanatorium Benefits—A Tragedy of Inexperience—The Protection of Advanced Consumptives in Poor-Law Institutions—The Appeal of Aberdeen Royal Infirmary—The Future of Farnfield Reformatory—The Resurrection of the Sunday Funds—A British Hospital at Limoges—Some Drawbacks—Infirmary Officers and Enlistment—Bradford War Hospital—Cavell Memorial Statue—The Hospital of Friendship—The War's Effect on Bequests to Charity—Badges for Pharmacists?—A Change of Policy—The Work of a Rural Medical Officer—A Hospital Secretary and the Navy—Hospital Committee and County Council: A Disputed Point—A Military Hospital Magazine—Salary or Fees—The Health of Halifax—Motor Ambulance for the Navy—This Week's Drug Market.

**At a Special Base Hospital** ... ... ... 161

How the R.A.M.C. Saves Combatant Wastage.

**A "Revolt" of Sanatorium Patients** ... ... 162

**The National Guard and the Hospitals** ... 163

St. Bartholomew's Hospital: Precautions Against Air Raids.

**Round the World** ... ... ... ... ... 164

Fellow-Workers and the Roll of Honour.

**Hospital Employees and Enlistment** ... ... 165

The Strain on the Institutions.

**The Use of Abbreviations in Prescribing** ... 166

**National Insurance Act Developments** ... ... 167

Effects of the Increase in Employment of Women.

PAGE

**Parliament and the Profession** ... ... ... 168

The Nurse's Wardrobe—An Alleged Wastage of Medical Men—Recovery of Fees from Doctors—Promotion of Army Doctors—Diseases in the Army—The Royal Army Medical Corps—The Treatment of Insured Consumptives—Maternal Mortality.

**Hospital Architecture and Construction** ... 169

Royal Victoria and West Hants Hospital.

**Buildings Contemplated** ... ... ... ... 169

**Brave R.A.M.C. Workers** ... ... ... ... 170

The D.C.M. for Field Ambulance Men.

**Emergency Military Hospital Construction** ... 170

**Personalia** ... ... ... ... ... ... 170

**Patriotism and the Nurse-Training Schools** ... 171

IV.—Some London Poor-Law Infirmaries: Edmonton Military Hospital—Hampstead Infirmary—Marylebone Infirmary—Whitechapel Infirmary.

**Passing Events and Topics** ... ... ... ... 172

No Red Cross—Alexandra Day in Leicester—A County Council Medical Committee—The German Doctors' Ten Commandments—French School Buildings as Hospitals—Medals for Nurses.

**Almeric Paget Massage Corps** ... ... ... 172

**Gifts and Bequests** ... ... ... ... ... 172

**The Institutional Worker** ... .. (Suppl.) 1

Dispensing Pupils: Answer to October Question.

Palatable Fare Without Meat.

Enquiries and Answers:

The Sick and In Need: Home for Infectious Case—Home for Epileptic Child—Home for Incurable.

The Housekeepers' Department: Newspaper Firelighters—Egg Substitutes.

Employment and Training: Private and District Institutions—Cottage Nursing—Dispensing at Military Hospitals—Health Visitors.

Practical Matters: *X*-ray Tubes.

